# Perioperative Management and Anesthetic Challenges of Abdominal Compartment Syndrome: A Case Report

**DOI:** 10.7759/cureus.93836

**Published:** 2025-10-04

**Authors:** Colette B Gazonas, Alejandro Hallo-Carrasco, Evan Berger

**Affiliations:** 1 Department of Medicine, Newark Beth Israel Medical Center, Newark, USA; 2 Department of Anesthesiology, Rutgers University New Jersey Medical School, Newark, USA; 3 Department of Anesthesiology, Newark Beth Israel Medical Center, Newark, USA

**Keywords:** abdominal compartment syndrome, abdominal decompression, anesthetic management, exploratory laparotomy, intraoperative hemodynamic instability, perioperative care

## Abstract

We present the perioperative management of a patient with abdominal compartment syndrome (ACS) secondary to fecal impaction and bowel perforation. This case highlights the complex challenges associated with ACS in the perioperative setting, particularly the high risk of cardiovascular and respiratory instability. Sudden hemodynamic collapse following induction or surgical incision, as well as complications during decompression, underscore the profound physiologic derangements inherent to this pathology and the critical role of anesthetic planning in patients with this presentation. Key anesthetic considerations include aggressive pre-induction fluid resuscitation, vasopressor preparedness, judicious selection of induction agents, and vigilant intraoperative monitoring of hemodynamic and respiratory status. Anticipatory planning is essential to optimize outcomes in these high-risk patients.

## Introduction

Abdominal compartment syndrome (ACS) is a life-threatening condition resulting from sustained intra-abdominal hypertension (IAH), typically characterized by an intra-abdominal pressure (IAP) exceeding 20 mmHg, which compromises blood flow and leads to progressive end-organ damage [1]. It often results in multi-organ dysfunction, most commonly affecting the renal, gastrointestinal, cardiovascular, and respiratory systems. Elevated IAP impairs venous return, reduces preload, limits ventilation, and reduces perfusion, necessitating meticulous perioperative management.

ACS is classified as either primary, resulting from intra-abdominal pathology such as trauma or bowel obstruction, or secondary, due to systemic factors like massive fluid resuscitation or sepsis. The interplay of both primary and secondary causes often complicates resuscitation efforts, underscoring the importance of early intervention and multidisciplinary coordination.

While the formal diagnostic criteria for ACS are beyond the scope of this case report [2], we present a complex and emergent clinical scenario of a 72-year-old male who developed ACS secondary to bowel obstruction and perforation and required urgent surgical intervention. We aim to highlight the perioperative challenges due to profound hemodynamic instability, respiratory compromise, and the risk of rapid deterioration from the anesthesiologist’s perspective.

## Case presentation

A 72-year-old male with class II obesity, hepatic steatosis, hypothyroidism, hypertension, and a history of alcohol and opioid use disorder (on 155 mg methadone daily) presented to the emergency department from his nursing facility with abdominal pain, distention, and no bowel movements for over three weeks. On arrival, he was alert, oriented, and able to provide a history of his present illness.

**Figure 1:**
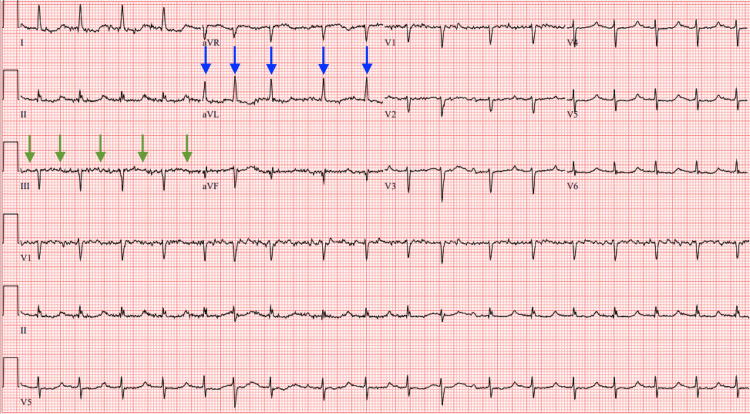
Electrocardiogram Electrocardiogram at time of admission showing atrial fibrillation with rapid ventricular response and evidence of age-indeterminate infarcts. Atrial fibrillation is evidenced by a lack of discernible P waves (green arrows) and irregularly spaced QRS complexes (blue arrows).

On admission, electrocardiogram revealed atrial fibrillation with rapid ventricular response and age-indeterminate infarcts (Figure 1). Initial laboratory studies were notable for elevated troponin suggestive of a type II myocardial infarction, anion gap metabolic acidosis, and acute kidney injury on chronic kidney disease. Additional findings included mild hyponatremia and evidence of systemic inflammatory response syndrome, raising concern for sepsis (Table 1).

**Table 1:** Labs of significance at time of admission MI: myocardial infarction; BUN: blood urea nitrogen; AKI: acute kidney injury; CKD: chronic kidney disease; eGFR: estimated glomerular filtration rate; HR: heart rate; SIRS: systemic inflammatory response syndrome; WBC: white blood cells.

Parameter	Patient Value	Reference Range	Units	Clinical Correlation	
Troponin	80	≤22	ng/L	Type II MI	
Anion gap	21	4-12	mmol/L	Anion gap metabolic acidosis	
Lactate	2.4	0.5–2.2	mmol/L	Anion gap metabolic acidosis	
BUN	47.8	10-26	mg/dL	AKI on CKD	
Creatinine	1.8	0.5–1.2, baseline 1.1	mg/dL	AKI on CKD	
eGFR	39.5	>60	mL/min/1.73 m²	AKI on CKD	
Sodium	133	136–145	mmol/L	Hyponatremia	
HR	112	60–100	beats/min	Meets SIRS criteria	
WBC count	17.2	4.0–10.5	×10³/µL	Meets SIRS criteria	

**Figure 2:**
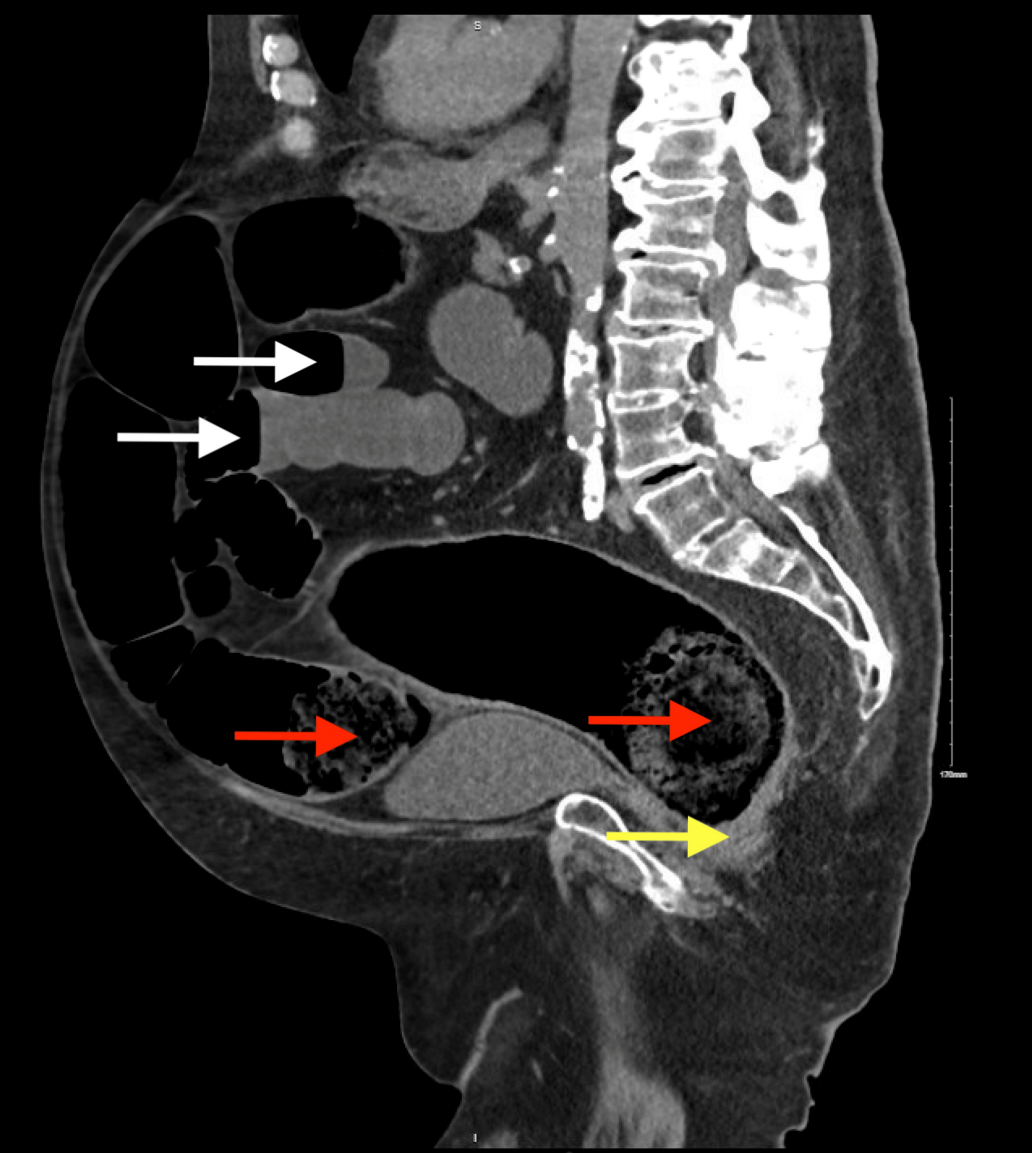
Computed tomography scan of the abdomen Computed tomography scan of the abdomen on hospital day 1 demonstrating severe fecal impaction (red arrows), stercoral colitis (yellow arrow), and multiple dilated loops of small bowel with air-fluid levels (white arrows).

A computed tomography (CT) scan of the abdomen on hospital day one demonstrated severe fecal impaction, stercoral colitis, and multiple dilated loops of small bowel with air-fluid levels, consistent with ileus or bowel obstruction likely secondary to high-dose opioid use (Figure 2). Although exam under anesthesia, colonoscopy, and possible exploratory laparotomy were recommended, the patient initially declined intervention and medical management was attempted. On hospital day two, repeat imaging revealed new-onset pneumoperitoneum, prompting emergent exploratory laparotomy, to which the patient consented.

The patient denied any history of anesthesia-related complications. Preoperative examination revealed a weight of 107.1 kg with a body mass index of 38.1 kg/m². Vital signs at that time were significant for hypertension and tachycardia. The patient’s temperature was low, but did not meet the criteria for hypothermia (Table 2). On exam, he was alert and oriented, cardiac rhythm was regular, and lungs were clear to auscultation bilaterally. Airway evaluation demonstrated full neck range of motion, good mouth opening, Mallampati score II, and thyromental distance greater than three finger breadths. The patient was edentulous, and upper and lower dentures were removed. Given the emergent nature of the procedure and high risk of mortality without surgical intervention, he was classified as American Society of Anesthesiologists (ASA) class 5E. Preprocedural concerns included a history of atrial fibrillation, chronic kidney disease, and pyelonephritis. His edentulous status was also identified as a potential challenge for facemask ventilation during induction.

**Table 2:** Preoperative and post-intubation vital signs BP: blood pressure; HR: heart rate; RR: respiratory rate; SpO_2_: oxygen saturation; T: temperature; ETCO_2_: end-tidal carbon dioxide.

Parameter	Preoperative Value	Post-intubation Value	Reference Range	Units
BP	154/82	82/68	(90-120)/(60-80)	mmHg
HR	120	109	60-100	beats/min
RR	20	10	20-Dec	breaths/min
SpO_2_	95	72	95-100	%
T (temporal)	35.8 (96.4)	-	36.1-37.2 (97-99)	°C (°F)
ETCO_2_	-	25	35-45	mmHg

Rapid sequence intubation was performed with rocuronium 100 mg, etomidate 20 mg, and propofol 20 mg. Immediately following uncomplicated intubation, the patient acutely decompensated, exhibiting hypotension, tachycardia, hypoxemia, and low end-tidal CO₂, with pallor and poor distal perfusion (Table 2). Endotracheal tube placement was confirmed, but oxygen saturation readings remained obtainable only at the nares.

Post-induction, invasive blood pressure monitoring via the left radial artery and central venous access via the right internal jugular vein were established. Midazolam 5 mg and sevoflurane 0.5%-0.6% were administered intraoperatively for maintenance anesthesia. Multiple doses of phenylephrine and ephedrine were administered in combination with aggressive fluid resuscitation (500 mL 5% albumin and 2,000 mL normal saline). Despite these interventions, the patient remained hypothermic and hemodynamically unstable, requiring triple vasopressor therapy: phenylephrine (300 µg), norepinephrine (4 µg/min), and vasopressin (2 units [0.1 mL]). In total, the patient underwent 89 minutes of anesthesia and 46 minutes of surgery. Urine output was approximately 400 mL, with an estimated blood loss of 50 mL. 

Although abdominal compartment pressures were not measured preoperatively or postoperatively, intraoperative findings were highly suggestive of ACS secondary to colonic perforation, with approximately 2 liters of fecal material in the abdominal cavity. The patient’s hemodynamics initially improved following abdominal incision, likely due to pressure relief. However, after resection of necrotic bowel and thorough irrigation of the peritoneal cavity with warm saline, the patient developed hypotension; the decision was made to leave the abdomen open and apply a vacuum-assisted closure (VAC) dressing. The decision was also made to leave the patient intubated and mechanically ventilated postoperatively. He was transferred to the intensive care unit (ICU) due to high risk of imminent, life-threatening deterioration. In the unit, he required vasopressor support in the form of norepinephrine and sedation with propofol (5-50 μg/kg/min) and fentanyl (25-300 μg/hr). 

Two days later, re-exploratory laparotomy was performed, during which multiple areas of gangrenous small bowel and colon were resected, and an end ileostomy with colonic mucous fistula was created. Anesthesia was induced with propofol (20 μg/kg/min; total 263.5 mg), rocuronium (50 mg), and fentanyl (75 μg/hr; total 153.8 μg). Intraoperatively, the patient received additional rocuronium (30 mg), fentanyl (100 μg), and sevoflurane (0.9%-2.5%) for maintenance of anesthesia. Intravenous fluids included 5% dextrose and lactated Ringer’s solution (150 mL/hr; total 307.5 mL). He remained unstable throughout the procedure, requiring vasopressor support with norepinephrine infusion (4μg-5 μg/min; total 518 μg). 

Hemodynamics fluctuated intraoperatively, with arterial blood pressure ranging from 106-212/37-88 mmHg, heart rate from 42-105 beats per minute, oxygen saturation from 87-100%, and temperature from 35.2-37.1℃ (95.4-98.8℉). Total anesthesia time was 123 minutes while surgical time was 84 minutes. Urine output totaled 100 mL, and estimated blood loss was minimal.

Postoperatively, the patient returned to the ICU intubated on pressure support ventilation with settings of pressure support 7 cm H₂O, tidal volume 500 mL, respiratory rate 12, and fraction of inspired oxygen 21%. He was successfully weaned off mechanical ventilation and extubated two days later. Vasopressor support and sedation were continued until postoperative day four. Norepinephrine was administered as a continuous infusion titrated to effect, peaking at 15 μg/min. Sedation was maintained with propofol (5-50 μg/kg/min) and fentanyl (25-300 μg/hr) infusions.

Due to persistent postoperative ileus, total parenteral nutrition (TPN) was initiated on postoperative day six. After eight days, he was transitioned off TPN and advanced to a pureed diet.

Antimicrobial therapy included a five-day course of intravenous piperacillin-tazobactam initiated on hospital day one, followed by metronidazole started one day after the first procedure. Following re-exploratory laparotomy, intravenous meropenem and micafungin were added on postoperative days three and six, respectively, per infectious disease recommendations.

Two weeks after extubation, the patient was downgraded from the ICU to the progressive care unit. The abdomen was not definitively closed during this admission. Five days later, he was discharged back to his nursing home with a wound vacuum device in place (with instructions for dressing changes every two to three days), a peripherally inserted central catheter for 20 additional days of intravenous meropenem and micafungin, and a 10-day course of oral linezolid suspension due to new bilious drainage from the VAC dressing concerning for recurrent intestinal perforation, with abdominal CT imaging suggestive of an abscess.

## Discussion

We present the perioperative management of ACS through the case of a patient with ACS secondary to fecal impaction in the setting of high daily opioid requirements. Careful preoperative planning and understanding of ACS pathophysiology are critical to reduce morbidity and mortality.

Prior to induction, optimization of cardiovascular and respiratory status is essential to mitigate the risk of rapid hemodynamic deterioration. In ACS, elevated IAP leads to increased airway pressures, reduced lung compliance, and impaired venous return, contributing to hypoxemia and circulatory instability. Thorough pre-oxygenation and apneic oxygenation via nasal cannula can help reduce the risk of rapid desaturation during apnea [3]. Given the loss of compensatory sympathetic tone and diminished preload, induction agents with favorable hemodynamic profiles, such as ketamine or etomidate, should be prioritized. While aggressive pre-induction fluid resuscitation can improve compromised venous return [4], the risks of fluid loading must be carefully weighed to avoid exacerbating IAH [5]. Pre-induction vasopressor support may also be considered [6].

Arterial and central venous access were not obtained in this patient until after induction, largely because ACS was not suspected preoperatively and the patient appeared clinically stable, masking the severity of his underlying condition. In any case of suspected or confirmed ACS, an arterial line should be placed prior to induction to allow for continuous blood pressure monitoring [7].

Post-induction, ACS compromises respiratory mechanics, as elevated IAP results in decreased pulmonary compliance, total lung capacity, functional residual capacity, and residual volume [8]. Thus, lung-protective ventilation strategies, such as conservative tidal volumes (6-8 mL/kg) and permissive hypercapnia, are recommended to prevent overdistention of the compressed thoracic cavity [9].

While surgical decompression can reverse respiratory failure from hypoventilation, it may also trigger abrupt physiologic shifts, including alkalosis, hypotension, and arrhythmias. These are thought to result from washout of tissue-toxic products of anaerobic metabolism and a rapid drop in systemic vascular resistance. In some cases, respiratory alkalosis may develop post-decompression in response to increased lung compliance and lung volumes, and a corresponding rise in minute ventilation [10]. The risk of these adverse events can be mitigated through vigilant respiratory monitoring, appropriate ventilator adjustments, and administration of a pre-decompression fluid bolus. Additionally, large-bore intravenous access and preparedness for massive transfusion are essential given the risk for significant blood loss and fluid shifts during decompression.

Timely initiation and completion of decompression are critical to minimize both the duration of elevated IAP and the cumulative exposure to anesthetic and operative stress. Given the hemodynamic instability of ACS patients, intervention should be performed expeditiously to limit exposure to the physiologic burden of surgical stimulation and anesthesia. Any post-induction delay in decompression places additional strain on an already critically ill patient and increases the risk of adverse outcomes.

## Conclusions

ACS is a rare but life-threatening condition that requires early recognition and carefully coordinated perioperative management. This case highlights the profound hemodynamic and respiratory instability that can occur with induction of anesthesia and surgical decompression in patients with ACS. Key anesthetic considerations include early invasive monitoring, pre-induction fluid resuscitation, vasopressor preparedness, and thoughtful selection of induction agents to maintain cardiovascular stability. Prompt surgical intervention and multidisciplinary coordination are essential to improving outcomes. Clinicians should maintain a high index of suspicion for ACS in patients with abdominal distention and multi-organ dysfunction, particularly in those with risk factors such as opioid-induced bowel dysfunction.
